# The case for rejecting the memristor as a fundamental circuit element

**DOI:** 10.1038/s41598-018-29394-7

**Published:** 2018-07-20

**Authors:** Isaac Abraham

**Affiliations:** DCG Silicon Development, DCG, Intel Corporation, Hillsboro, OR USA

## Abstract

The memory resistor with the moniker memristor was a harmless postulate in 1971. Since 2008 a device that claims to be the memristor is on the prowl, seeking recognition as a fundamental circuit element, sometimes wanting electronics textbooks to be rewritten, always promising remarkable digital, analog and neuromorphic computing possibilities. A systematic discussion about the fundamental nature of the device is almost absent within the memristor community. Advocates use incomplete constitutive relationships, ignore concepts of activity/passivity and aver that nonlinearity is central to their case. Few researchers have examined these claims. Our report investigates the assertion that the memristor is a fundamental passive circuit element, from the fresh perspective that electrical engineering is the science of charge management. We demonstrate with a periodic table of fundamental elements that the 2008 memristor is not the 1971 postulate and neither of them is fundamental. The ideal memristor is an unphysical active device and any physically realizable memristor is a nonlinear composition of resistors with active hysteresis. We also show that there exists only three fundamental passive circuit elements.

## Introduction

The basic question of the “missing circuit element” is whether we can we have a new passive element that cannot be made from the combination of existing passive elements. The capacitor (*C*), resistor (*R*) and inductor (*L*) are the three fundamental passive elements that a contemporary electrical engineer is familiar with. The word fundamental means fundamental passive in the rest of this document. We start the study by generating charge-voltage relationships for *C*, *R* and *L*. Then we attempt to integrate the memristor into the mix.

**Figure 1:**
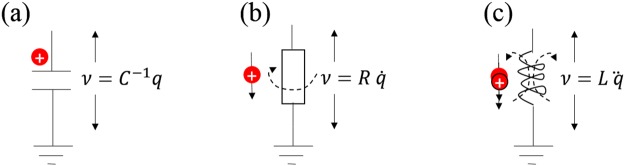
Fundamentals of electrical engineering in the charge-voltage perspective. (**a**) Charge stored on a capacitor generates voltage *ν*. (**b**) A rate of charge (current) through a resistor generates voltage *ν* and magnetic field. (**c**) Rate of rate of charge (di/dt) in an inductor generates voltage *ν* and a magnetic field. Rate of rate is indicated by the double charge-and-arrow.

Figure 1 represents the essence of electrical engineering (EE). For clarity in discussion, we assume that non idealities like leakage, temperature, noise or voltage coefficients are absent. Only the phenomenon being discussed is in effect. While units may not always be explicitly stated, we assume SI units^1^. In Fig. 1, charge is shown as a red dot with the inset plus sign, and magnetic fields where they exist are shown as dashed, curved arrows. Throughout the report, we use lower case symbols for charge (*q*), voltage (*ν*) etc. to allow representation of the most general cases. Phenomenological constants are in upper case. Table 1 introduces concepts that appear in this report and list some example devices that conform to the concepts. We follow the guidance that a regular (non-Esaki) pn diode is a nonlinear passive element because it does not amplify power^2^.

**Table 1:** Definition of terminology.

#	Concept	Description	Example Devices
1	Linear	Linear implies that a doubling of the input signal produces a doubling of the output signal.	*C*, *R*, *L*.
2	Nonlinear	Description 1 does not apply.	Diode, transistor.
3	Active	A physical device that can produce power gain.	Transistor.
4	Passive	A physical device that cannot produce power gain.	*C*, *R*, *L*, diode, switch.
5	Composite	A device that can be modeled from fundamental components.	*R* ± *jX*, potentiometer.
6	Fundamental	Irreducible electrical representation of linear, passive elements.	*C*, *R*, *L*.

A frequent situation in EE is that, in a non-zero electrical field, a charge is separated from its reference plane by applying some energy. This results in a static charge placed at some location, with a potential. The potential is the work that will be done by this charge as it travels back to its reference, with units joules/coulomb or volt. This is naturally visualized by an electrical engineer as a capacitor storing charge. The charge is held immobile and the voltage across the capacitor is *ν* = *C*^−1^*q*, where *ν* is voltage, *q* is charge and *C* is capacitance. Capacitance *C* is the phenomenological constant with the unit of farad (F) that translates charge to voltage. This is shown in Fig. 1(a).

In classical mechanics, a frequent situation is for an object to move with a constant velocity in a viscous media, implying friction and energy dissipation. For a charge, the equivalent case is to flow through an element with non-zero electrical field, in this case a resistor. This is depicted in Fig. 1(b). The rate of charge is current. The governing equation is $\nu =R\,\dot{q}$, which is the familiar *ν* = *iR* where $i=\dot{q}$. The over-dot in $\dot{q}$ represents derivative with respect to (w.r.t) time. The phenomenological constant is resistance *R* with the unit ohm (Ω).

In classical mechanics, acceleration is the rate of change of velocity. Similarly when current changes during its travel from location A to location B, it is a rate of rate of charge; equivalent to the second derivative of charge w.r.t time. The governing equation for the element that can convert a rate of rate of charge into voltage is $\nu =L\ddot{q}$. The phenomenological constant is inductance *L* with the unit of henry (H). We are more used to the standard form $\nu =L\frac{di}{dt}$^2,3^. We show this in Fig. 1(c).

Using the information above we generate a sketch of the periodic table of fundamental elements in the charge-voltage domain. We have borrowed the expression “periodic table” as attributed to Chua^4^. A periodic table of fundamental elements is the very tool that is used by proponents to market the memristor as a fundamental element.

## Results

### The periodic table of fundamental passive elements

**Figure 2:**
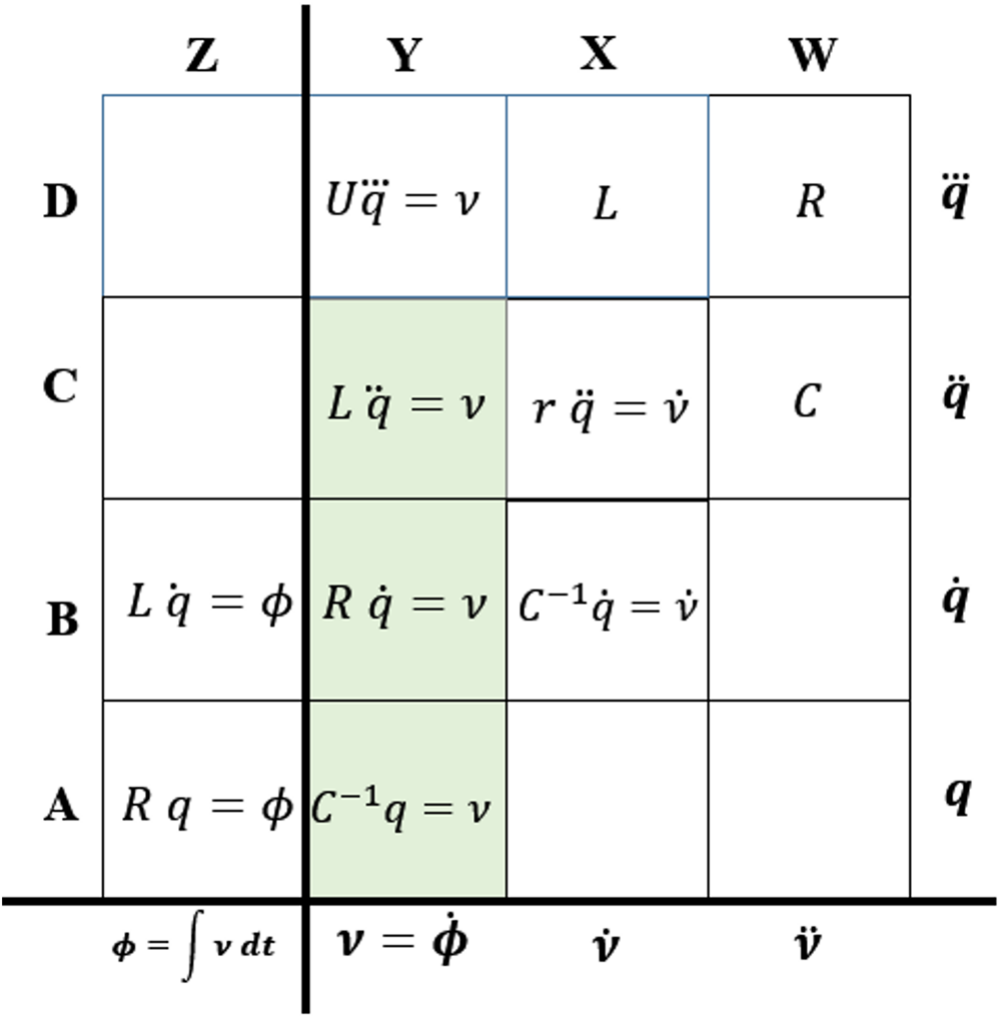
The periodic table of fundamental passive elements in the charge-voltage domain.

The periodic table in Fig. 2 has rows and columns of the grid labeled in upper case alphabets along the left and top edges. We will address a grid by its (row, column) label. The actual electrical variable that applies to each row or column is shown along the right and bottom edge. On the horizontal axis each column to the right is a time derivative of the one on its left. For example the x axis of (A, Y) is $\dot{\varnothing }$ which is the derivative of *ϕ*, the x axis of (A, Z). Similarly along the y axis, each higher row’s y axis is the derivative of the one below it.

Existing fundamental elements satisfy the following rules. We expect the same compliance from the memristor.

(i) Rule 1: Only one fundamental element can occupy a slot in the periodic table.
(ii) Rule 2: A transient event will not count as satisfying a constitutive relationship.

Rule 1 takes guidance from the periodic table of chemical elements which organizes elements based on atomic number. The periodic table of electrical elements organizes its elements based on the n^th^ derivative of charge that relates the phenomenological constant to the voltage developed across the device.

Rule 2 is inferred from the definition of existing fundamental elements namely *C*, *R* and *L*.

### Locating the fundamental elements

The known fundamental elements from the preceding discussion appear along column Y rows A, B and C. This placement was done by comparing the governing equation of the slots to the governing equations in our earlier discussion. Moving into column X, slot (A, X) is empty because there is no known element that satisfies its rule. Slot (B,X) satisfies the derivative based definition of a capacitor namely $C\frac{d\nu }{dt}=i$ or the form that maps to our periodic table ${C}^{-1}\dot{q}=\dot{\nu }$. Similarly in column X, we observe that (C, X) is $r\,\ddot{q}=\dot{\nu }$ which is the small signal definition of a resistor, equivalent to $r=\frac{d\nu }{di}$. Inspection shows that each of the known fundamental elements travels along diagonals in our periodic table, leading to the n^th^ derivative representation in terms of charge and voltage.

The thick vertical separator between columns Z and Y reinforces the idea that fundamental elements do not percolate into column Z. Columns to the right of Y contain derivatives of the governing equations from Y and do not constitute a fundamental definition because they are just mathematical operations. If there is place for a new fundamental element, it would be slot (D, Y) with a governing relation $U\,\dddot{q}=\nu$, equivalent to $\nu =U\frac{{d}^{2}i}{d{t}^{2}}$, where *U* is some yet to be discovered phenomenological constant. However, we will demonstrate later that an occupant of (D, Y) will be active, hence neither passive nor fundamental.

Before discussing column Z, let us review the governing equation that relates voltage to magnetic flux; namely Faraday’s law. Faraday’s law states that the negative of the rate of change of magnetic flux (${\varnothing }_{B}$) will be equal to the electric potential (∈) developed in the element, such as an inductor.

1 $${\epsilon }=-\,\frac{d{\varnothing }_{B}}{dt}$$

Assume that the experimenter is forcing a change in magnetic flux. The negative sign implies that the resulting voltage will be in such a direction as to generate a current whose magnetic field will try to oppose the forcibly induced change in flux. The equation is intended for use in a situation where the flux is truly a magnetic flux. However we notice that we could integrate the left hand side of equation (1) to result in $\varphi =-\,\int {\epsilon }\,dt$ without insisting on a magnetic field. We have used *ϕ* without the subscript to denote the computed flux rather than the real magnetic flux. While this approach is mathematically correct, it gives rise to a possible misuse of the term flux. The 1971 postulate vacillates by employing both *ϕ* (time integral of voltage) and the words *flux-linkage* (used therein seven times), suggesting that the originally postulated memristor concept did indeed hinge on the existence of magnetic flux^5^. Given that we already have a charge-voltage plane to inspect, let us accommodate the definition $\varphi =-\,\int {\epsilon }\,dt$ and disregard the need for a magnetic flux.

This moves our discussion into column Z in Fig. 2. All elements along column Z generate flux $\varphi =\int \nu \,dt$. Here $\nu \equiv {\epsilon }$ and represents voltage (electric potential). The missing negative sign (w.r.t a magnetic context) only serves to dictate the direction of voltage and there is no loss of accuracy for the discussion by leaving it out. Of the three known fundamental elements, only the inductor and resistor appear in column Z. As for the capacitor, we could mathematically write $\varphi ={C}^{-1}\int q\,dt$ and be correct. However this would put us in a *meaningless* slot below (A, Z). In this system with passive elements, the capacitor did not make it into column Z because charge on a capacitor cannot be meaningfully transformed into the integral of voltage. This example with the capacitor shows how to create an infinite grid periodic table.

Turning our attention to slot (A, Z), the generalized governing equation is $U\,q=\varnothing$. We have temporarily introduced the phenomenological constant *U* to stand in for what we might discover. Simple transposition gives $U=\frac{\varnothing }{q}$. We can rewrite this based on its time derivative form where the over-dot represents derivative w.r.t time.

2 $$U=\frac{\dot{\varnothing }}{\dot{q}}=\frac{d\varphi }{dq}=\frac{\nu }{i}$$

Equation (2) defines the resistor, making *U* in (A, Z) equal *R* as shown in Fig. 2. Prodromakis *et al*. among others use the intermediate expression $(\frac{\frac{d\varnothing }{dt}}{\frac{dq}{dt}})$ containing the reference to charge, to suggest that *U* = *M* = *M*(*q*(*t*))^6^. There are two uncomfortable issues here.

(i) While the inference is not wrong, it violates Rule 1 because the simple resistor will also suitably occupy slot (A, Z).
(ii) Any phenomenological constant inferred from time dependent intermediate equations is tenuous and violates Rule 2.

### Comparison with Strukov’s table of fundamental elements

**Figure 3:**
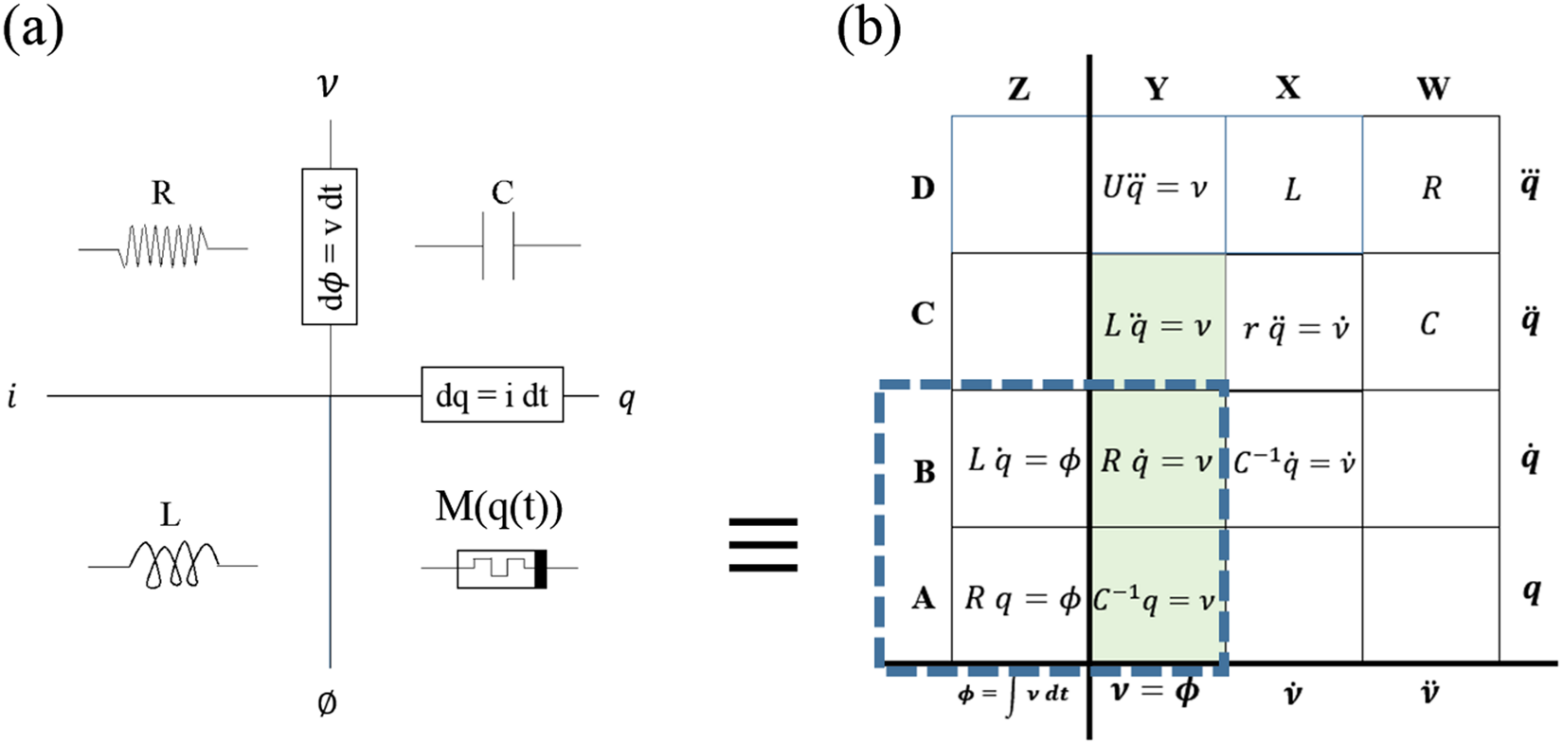
Chart of fundamental passive elements in the charge-voltage domain. (**a**) Strukov’s interpretation attributed to Chua. (**b**) Our representation shows Strukov’s chart as a subset enclosed within the blue dashed rectangle.

Figure 3 compares the periodic table of fundamental elements published by Strukov *et al*. at Hewlett Packard (HP) with that proposed by this paper^7^. We see that a left ninety degree rotation on the grid (A, Z)-(B, Y) in Fig. 3(b) definitely makes it resemble Strukov’s chart. There is a mistake in that original chart of fundamental elements^7–9^. The framed expression on the positive x axis, *dq* = *idt* conflicts with the positive x axis being simultaneously labeled *q*. Algebraic manipulation of the framed expression gives $\frac{dq}{dt}=i$; implying that current equals the charge label along the positive x axis. This may be a typographical error and is not observed to have affected any inferences because the expression is always overlooked. Nalawade *et al*. have corrected this but manage to label the flux axis incorrectly^10^. Kvatinsky *et al*. simply retain the empty frames^11^. Kumar has correctly labeled the chart’s axes in a review^12^.

From the side by side comparison in Fig. 3, we observe that the lower part of Strukov’s chart maps to our column Z in Fig. 3(b). Occupants of column Z are not fundamental because their relationships are defined by mathematical integration, requiring initial conditions. Even if we allowed such elements into the fundamental fold, the position proposed for the memristor is already occupied by the resistor.

The charge-voltage plane is sometimes referred to as the charge-flux domain, which is just the same as Fig. 2, including the forbidden column Z. The memory resistor of 2008 with its phenomenological constant *M*, does not find a place in the charge-voltage plane because of the following two reasons.

(i) Slot (A, Z) is already occupied by the resistor. Rule 1 prohibits a second occupant.
(ii) A memristor with $M(q(t))=\frac{\varnothing }{q}$ can only be evaluated by integration, violating Rule 2 by requiring a time-window for the integral.

### The circuit-theoretic periodic table of passive elements

A moving charge generates a magnetic field. Ampere’s law can be used to deduce that a current carrying wire will produce magnetic field lines perpendicular to the wire, in the direction suggested by the right hand rule. This means that Fig. 1(b) and (c) have magnetic contributions as marked by the dashed curved arrows. This motivates us to expand the periodic table of fundamental elements into the magnetic plane.

**Figure 4:**
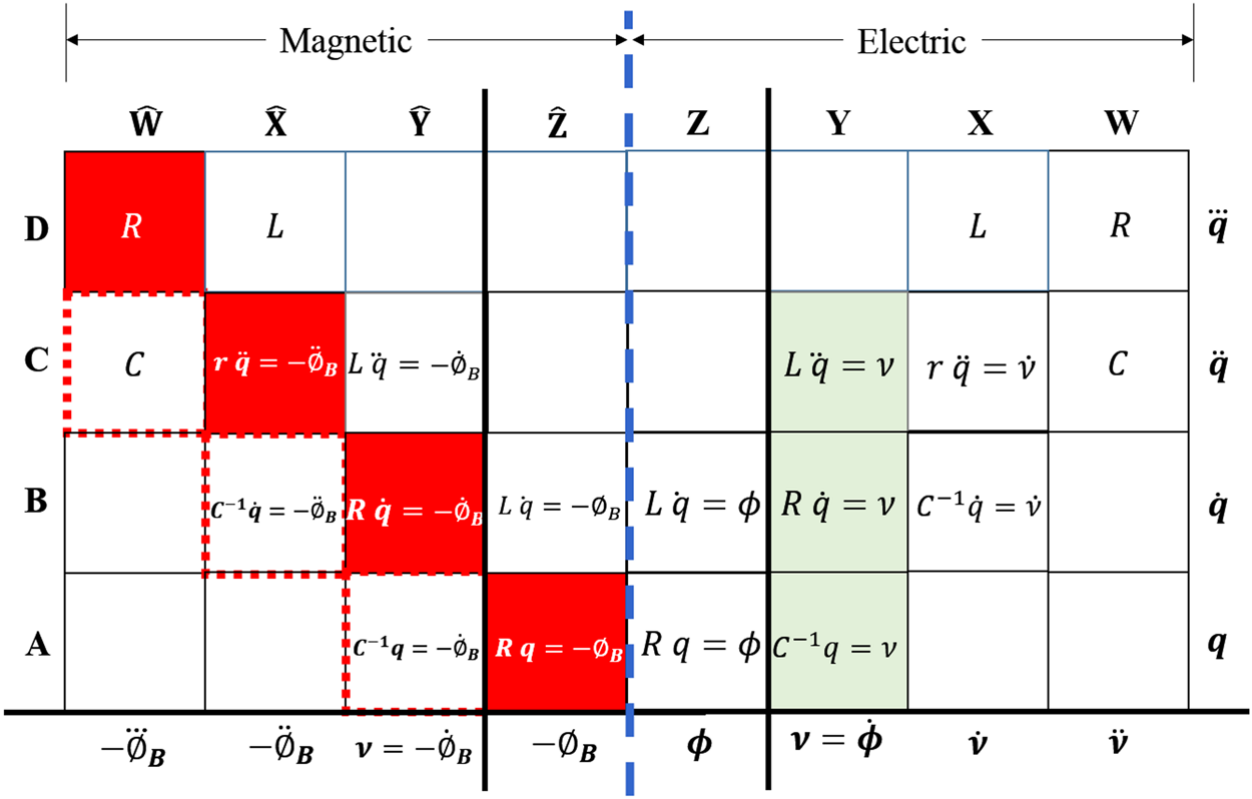
Periodic table of fundamental elements in the charge-flux (magnetic) and charge-voltage domains.

Figure 4 is the extended version of Fig. 2, with the charge-voltage plane to the right of the dashed blue center spine and the charge-flux (magnetic) plane to the left of the dashed blue center spine. The flux on the magnetic side is the true magnetic flux represented by *ϕ*_*B*_. Charge and its derivatives are along the y axis. The columns are labeled with hats on the magnetic side; row designators are shared among both domains. Discussion will address slots in (row, column) style. All devices within columns Z and $\hat{{\rm{Z}}}$ needed initial conditions to be specified to their derivative forms from Y and $\hat{{\rm{Y}}}$. To the left of center, each column leads into the n^th^ derivative of the true magnetic flux. To the right, each column similarly leads to the n^th^ derivative of computed flux, defined as the integral of voltage w.r.t time. Fundamental elements are in light green boxes that represent their constitutive relations. We start the discussion with the well-known candidates.

The capacitor from the charge-voltage plane does not make an appearance on the magnetic side because there is no magnetism involved for stationary charges. This automatically eliminates all the magnetic diagonals with capacitance *C*, like $({\rm{A}},\hat{{\rm{Y}}})$, $({\rm{B}},\hat{{\rm{X}}})$, $({\rm{C}},\hat{{\rm{W}}})$ and so on shown in red dotted boxes.

With respect to inductors, we notice immediately that the inductor exists as it should in slot $({\rm{C}},\hat{{\rm{Y}}})$. The inductor continues into slot $({\rm{B}},\hat{{\rm{Z}}})$, crosses into the electric domain at (B, Z) and further into slot (C, Y). The inductor can live in both planes. An inductor generates a voltage proportional to the rate of change of current.

3 $$\nu =L\,\ddot{q}$$

The inductor also creates a true magnetic field around the coils for alternating current conditions in $({\rm{C}},\hat{{\rm{Y}}})$.

4 $$-{\dot{\varphi }}_{B}=L\,\ddot{q}$$

Under direct current conditions, the inductor satisfies the following relationship in $({\rm{B}},\hat{{\rm{Z}}})$ and (B, Z).

5 $$-{\varphi }_{B}=L\,i=\varnothing $$

We recognize that $({\rm{B}},\hat{{\rm{Z}}})$ is a one way relationship where a constant current can produce a constant magnetic field but not vice versa. Similarly $L\,i=\varnothing$ in (B, Z) requires integration. Therefore equation (5) is not the constitutive relation for an inductor. Equation (4) from slot $({\rm{C}},\hat{{\rm{Y}}})$ or equation (3) from (C, Y) is the true constitutive relation for the inductor because it alone describes the ability of the inductor to bridge the magnetic and electric domains^2,3^.

Let us now presume that the original postulate about a magnetic memristor is true. This will mean that the device should occupy slot $({\rm{A}},\hat{{\rm{Z}}})$ based on the constitutive relation $M=-\,\frac{{\varnothing }_{B}}{q}$, with proper sign and subscripting. At this point we don’t have enough data to contest this existence. We know from the loci of fundamental elements, that an incumbent in $({\rm{A}},\hat{{\rm{Z}}})$ must also live in $({\rm{B}},\hat{{\rm{Y}}})$. The governing equation for slot $({\rm{B}},\hat{{\rm{Y}}})$ is $U\,\dot{q}=-\,{\dot{\varphi }}_{B}$. This rule requires that there is some device with phenomenological constant *U* which will produce a magnetic field that changes at a constant rate when a constant current ($\dot{q}$) is passed through it. Consider the 2008 HP (or 1971 Chua’s) memristor for this slot. Pushing a constant current into the memristor will cause the voltage across the device to change $(\dot{v})$ as its resistance changes. The constant stimulus current will however only create a constant magnetic field, definitely not $-{\dot{\varphi }}_{B}$. Therefore the 2008 HP (and 1971 Chua’s) device cannot occupy $({\rm{B}},\hat{{\rm{Y}}})$ except for the trivial condition $-{\dot{\varnothing }}_{B}=0$. A resistor also satisfies this trivial condition. Let us try to derive the rule for the observed $\dot{\nu }$ from basic laws. Differentiating Faraday’s law w.r.t time gives $\frac{d}{dt}{\epsilon }=-\,\frac{d}{dt}(\frac{d{\varphi }_{B}}{dt})$, which is $\dot{\nu }=-\,{\ddot{\varphi }}_{B}$. We see $-{\ddot{\varphi }}_{B}$ in slot $({\rm{C}},\hat{{\rm{X}}})$. This requires a device to generate a rate of rate of magnetic flux when a rate of current passed through it i.e. $r\frac{di}{dt}=-\,{\ddot{\varphi }}_{B}=\frac{dv}{dt}$. In other words, pushing a rate of current into the device should result in a rate of change of voltage $\dot{\nu }$; which will occur across the 2008 HP and 1971 Chua’s devices. However, the change of voltage is only momentarily nontrivial and will evaluate to zero as soon as the transition from low to high resistance (or vice versa) is over; making this a transient event and violating Rule 2. Therefore there is no place in $({\rm{C}},\hat{{\rm{X}}})$ for the 2008 (or 1971) memristor. By inference, if $({\rm{C}},\hat{{\rm{X}}})$ and $({\rm{B}},\hat{{\rm{Y}}})$ are devoid of memristors, then $({\rm{A}},\hat{{\rm{Z}}})$ is also forbidden to the memristor. Additionally, there is no incumbent possible in $({\rm{A}},\hat{{\rm{Z}}})$ because stationary electric charges cannot produce a magnetic field. Thus we have colored all the excluded squares of the periodic table in red, eliminating the 2008 HP (and 1971) device from the magnetic plane. Proposing that *q* in $({\rm{A}},\hat{{\rm{Z}}})$ is the integral of current and not a literal stationary charge, puts us in the realm of abstraction-by-integration. Then the memristor is an abstract device that responds to an abstract electric charge. In any case, a resistor already satisfies the conditions of $({\rm{A}},\hat{{\rm{Z}}})$ even in this realm of abstraction.

Can the 1971 postulate be probable *anywhere* in the magnetic side? The loci of fundamental elements suggests that nothing other than a form of resistor can occupy the trajectory $({\rm{A}},\hat{{\rm{Z}}})$, $({\rm{B}},\hat{{\rm{Y}}})$, $({\rm{C}},\hat{{\rm{X}}})$, $({\rm{D}},\hat{{\rm{W}}})$ etc. proposed for the memristor. The memristor in any manifestation is therefore excluded from the magnetic and electric side of the periodic table by Rule 1 and/or Rule 2.

### General opposition to the memristor concept

Vongehr *et al*. point out the problems with mixing *ϕ*_*B*_ and *ϕ*^13^. For them, the absence of magnetism in the 2008 HP model is its primary disqualifier and opine that if the memristor sans magnetism is valid, then we have also discovered an inductor that works without magnetism. Di Ventra and Pershin interpret mem-devices as response functions. They offer the possibility of obtaining current-voltage curves that do not cross the origin^14^. This is at odds with Chua’s definition which says that if its pinched it’s a memristor^15^. Chua states very clearly that “In this paper any two terminal black box is called a *memristor* if, and only if, it exhibits a *pinched hysteresis loop* for *all bipolar periodic* input current signals (resp., input voltage signals) which result in a *periodic* voltage (resp., current) response of the same frequency, in the voltage–current (*v* − *i*) plane”^15^. Sundqvist *et al*. study the memristor with thermodynamics considerations and conclude that the memristor equations are physically incomplete w.r.t to passivity or activity^16,17^. They object to the claim that the existing contender for the memristor is a passive device because it violates the second law of thermodynamics in an infinitely large number of cases while no positive example can be identified due to the unphysical character of Chua’s fundamental memristor definition. Similar criticisms about violation of Landauer’s principle, the absence of magnetic flux, changing definitions etc. are presented by Jeltsema^18^. The common denominator for objections converges on the absence of magnetism and the likely need for active elements to be present.

### The linear-nonlinear debate

The notion that the memristor postulated in 1971 may somehow exist as a nonlinear yet fundamental entity can be dispelled by reviewing Chua’s seminal paper. Section III of Chua’s paper states that when the memristor *ϕ*-*q* curve is a straight line, the memristor reduces to a *linear time-invariant resistor*; very much in keeping with our findings in the prior sections^5^.

Now consider the nonlinear case. Chua states that “…only memristors characterized by a monotonically increasing *ϕ*-*q* curve can exist in a device form without internal power supplies.” A nonlinear *ϕ*-*q* curve will always have a positive, albeit variable slope. However, the ratio of *ϕ* to *q* still has the units of ohm, *without a phase shift*, making this a nonlinear resistor. Simple pn diodes or transistors as shown in Table 1 can emulate nonlinear resistors. In the context of the three known fundamental devices, Tour *et al*. state that “The behavior of each of these elements is described by a simple linear relationship…”, clearly affirming that linearity is central to being fundamental^8^. Nonlinear resistors are not fundamental because they can be modeled by an assembly of piecewise linear components.

The report from Di Ventra *et al*. and general modeling knowledge shows that only an active element can produce a negative differential resistance (NDR) which is seen in the memristor current-voltage curves^14^. NDR eliminates the nonlinear passive diode from being able to model the memristor, leaving active circuits as the only option to model memristors.

Let us review the memristor in the light of Strukov’s expression for the phenomenological constant $M(q(t))=\frac{\varnothing }{q}$. When *M*(*q*(*t*)) is a positive constant and $\frac{d}{dq}M(q(t))=0$, then the device is a *linear time-invariant resistor* which is the already known fundamental element *R*. All other cases are at the very least nonlinear and excluded from being fundamental. By virtue of nonlinearity and *ignoring* any activity criterion, the memristor should occupy (C, X) in Fig. 4.

The suggestion that a circuit element can be a fundamental passive by virtue of nonlinearity is fallacious. If this were true, then the small signal resistor *r* in (C, X), which could represent a passive diode, would also be a fundamental element. Even if we choose to henceforth recognize nonlinear passive devices as fundamental in some “expanded design space” as suggested by Williams *et al*., (i) the memristor cannot occupy (A, Z) or $({\rm{A}},\hat{{\rm{Z}}})$ because those slots are occupied by the linear resistor and (ii) the memristor cannot occupy (C, X) because in the current-voltage domain the device exhibits hysteresis – an active phenomenon, thereby excluding it completely from any table of fundamental passive elements^5,7,19^. Slot $({\rm{C}},\hat{{\rm{X}}})$ was rejected in previous discussion.

### Extending the scope of the periodic table

With a well-developed tabulation of concepts in Table 1, a periodic table and two concise rules at our disposal, we are well equipped and inclined to review other forms of devices like the flux controlled inductor, charge controlled capacitor etc.

For example, consider a device that satisfies the relation $i=f({\varnothing }_{B})$ where we are assuming that flux ∅_*B*_ is magnetic flux. Any inductor will develop a voltage across the device terminals with constitutive relation $L\frac{di}{dt}=\nu$ in (C, Y). Substitute for *i* in the constitutive equation, after generalizing all quantities to be dependent on flux. We get $L({\varnothing }_{B})\frac{d\,f({\varnothing }_{B})}{dt}=\nu ({\varnothing }_{B})$, therefore $L({\varnothing }_{B})=\frac{\nu ({\varnothing }_{B})}{\dot{f}({\varnothing }_{B})}$. The device is fundamental iff *L*(∅_*B*_) is a positive constant and $\frac{d}{d{\varnothing }_{B}}L({\varnothing }_{B})$ is identically zero. All other cases with positive non-zero slope are nonlinear and fit into slot (D, X) which is the small signal definition of the inductor stated as $\frac{d}{dt}\nu =L\frac{{d}^{2}}{d{t}^{2}}i$.

As another example, consider a device with *ν* = *g*(*q*). All capacitors respond to charge and produce a voltage according to *Cν* = *q*. Reformatting the constitutive relation to fit our test case, we have *C*(*q*)*g*(*q*) = *q*. Transposing, $C(q)=\frac{q}{g(q)}$. The device is fundamental iff *C*(*q*) is a positive constant and $\frac{d}{dq}C(q)$ is identically zero. All other cases with positive non-zero slope are nonlinear and fit into slot (B, X) which is the small signal definition of the capacitor namely $\frac{d}{dt}\nu ={C}^{-1}\frac{d}{dt}q$.

In retrospect, we observe that column X contains the small signal representation of fundamental elements, just as column Y alone contains the constitutive relations of fundamental elements.

### What is Chua’s or HP’s memristor

If the memristor is not fundamental, then we strive to understand what it is. The first evidence is the original model from HP. It looks like a potentiometer made of two resistors and a slider^7^. The slider must be moved as a function of time to make the device transition between the low and high resistance states. In spite of the many shortcomings of the HP model it captures the essence of the memristor – a two terminal series connection of resistors with a low resistance *R*_*LO*_ and high resistance *R*_*HI*_, exhibiting NDR. While Di Ventra *et al*. argue that a negative resistance can only ensue from an active element, the memristor community does not readily equate NDR with the presence of an active element^14^. Through the clever use of window functions that are arbitrarily introduced into equations, HP hides the presence of active elements in their memristor model. After all, a potentiometer has no inherent hysteresis.

An original research in symbolic modeling has revealed two impedances that torsion in the complex plane^20,21^. That approach proposes the logistic function as the solution to a variable coefficient Burgers’ equation. The Burgers’ model reveals the memristor as the sum of a real and negative impedance; where the reactive components always sum to zero. The complex resistors have the form ${R}_{1}=\pm \,a\mp j\,b$ and ${R}_{2}=\mp \,c\pm j\,b$, where the positive term from among *a*, *c* is always larger. Therefore the composite resistance is always positive. The model unambiguously reveals the presence of a non-dominant negative resistance. It is possible to associate the negative impedance with a shockwave that Tang *et al*. have deduced^22^. The negative resistance is not visible to an external observer except during transition and is *indispensable* for representing flux dependent hysteresis which is key to memristor functionality. Without hysteresis, memristor current-voltage curves cannot exhibit lobes. The current-voltage curve with pinched hysteresis is a signature of the memristor^15^. Abraham’s symbolic model generates traditional current-voltage curves and exhibits a reasonable match to empirical switching time data^21^. The said model has also been demonstrated to exhibit correct temperature dependence w.r.t empirical data^20^.

Vacancy migration in a memristor is like the bubbles in a glass analogy by Williams, or rather like the behavior of devices with space-charge limited currents where the thermal relaxation time-constant of the space-charge is long^19^. The resulting boundary between the low and high vacancy concentration regions in the memristor emulates the slider of a rheostat, partitions the device into two (series) resistors and implements (active) hysteresis. Hysteresis is acknowledged by many authors including Chua, Williams, Strukov and Biolek^5,7,15,19,23^. Hysteresis can be implemented in circuit with the operational amplifier, Schmitt trigger or voltage/current-controlled elements – all of which are active.

The Chua Memristor Center’s website claims that Corinto *et al*. have constructed a memristor model with one port passive components^24,25^. This is impossible if the memristor is a fundamental element. Another peer reviewed publication associated with the circuit replaces the purported passive resistor with a Chua’s diode; which is a locally active device^26^. Local activity implies a negative resistance^27^. Memristor modeling has always needed active elements because it is implicitly active^5,28,29^.

**Figure 5:**
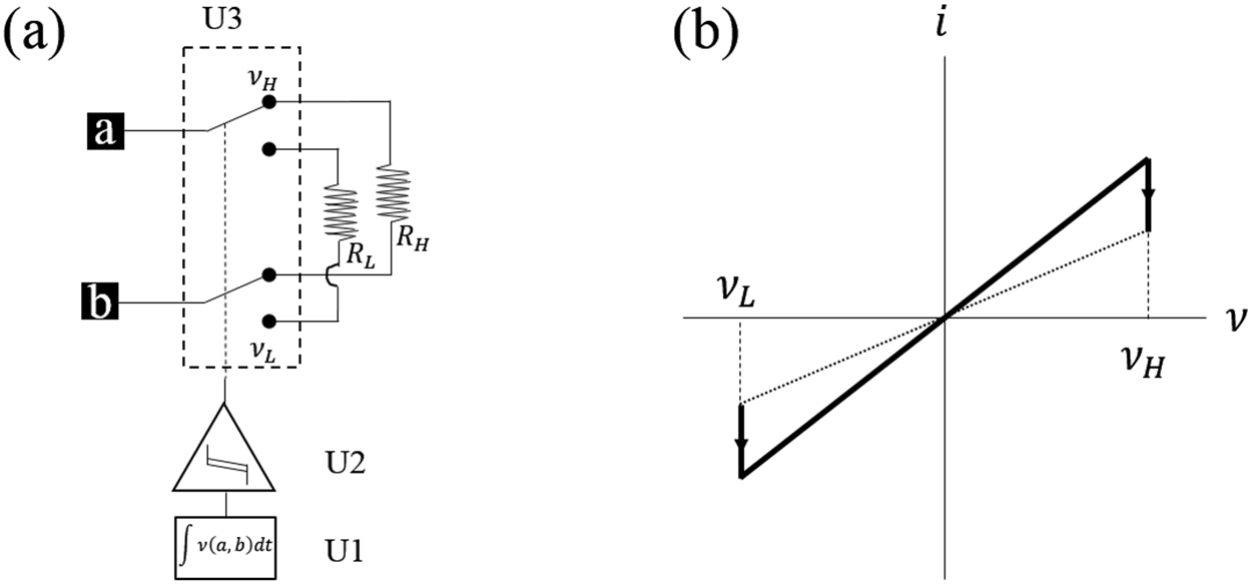
Memristor circuit model with flux based hysteresis. (**a**) Hysteresis is necessary to correctly select the low or high resistance at a predetermined flux threshold. (**b**) Current-voltage curve with hysteresis marked in the voltage domain.

In Fig. 5 we model the memristor as two resistors selectable with a double pole double throw switch U3 controlled by the hysteresis generator U2. The device terminals are *a* and *b*. The VTEAM model has a positive and negative threshold which could be produced by U2^30^. Integrator U1 computes flux. The hysteresis generator U2 is the unavoidable active component.

The Chua Lectures, Part 3 demonstrates that the ideal memristor will draw infinite current $i(t)={3\nu {(\varnothing (0)+\nu t)}^{2}|}_{t\to \infty }\to \infty$ for any non-zero voltage stimulus. The square law relationship w.r.t time hints at an unphysical active element^31^. This is remarkable because none of the other three fundamental elements are unphysical. A device with $i(t)\propto {t}^{2}$ could in theory occupy (D, Y) in Fig. 4. The “Locating the fundamental elements” section had suggested that (D, Y) *may* harbor a new fundamental device. Our mystery device could in theory be multiplying two inductor currents, each of $i(t)={L}^{-1}\nu \,t$. Multiplication however requires active elements thereby eliminating passive devices from ever occupying (D, Y). Therefore the set of fundamental elements is strictly limited to *C*, *R* and *L* along ([A, B, C], Y) in Fig. 4.

We summarize our findings in Table 2 where we list each fundamental element and its constitutive relation in the electric and magnetic planes. The variable *ν* represents voltage (V) and *ϕ*_*B*_ represents true magnetic flux in weber (Wb). Referring back to Fig. 4, we observe that in the electric domain, voltage is the measureable quantity that is generated across the terminals of fundamental elements in column Y. By virtue of symmetry, rate of change of magnetic flux $-{\dot{\varphi }}_{B}$ is the only measurable quantity that should help locate a fundamental element in the magnetic domain in column $\hat{{\rm{Y}}}$.

**Table 2:** Table of constitutive relationships for fundamental elements.

**#**	Device	*ν*	${\boldsymbol{-}}{\dot{{\boldsymbol{\varphi }}}}_{{\boldsymbol{B}}}$	Notes
1	Capacitor	*C* ^−1^ *q*	NA	A capacitor has no interaction with the magnetic field.
2	Resistor	$R\,\dot{q}$	NA	A current in the resistor can generate a magnetic field but not vice versa.
3	Inductor	$L\,\ddot{q}$	$L\,\ddot{q}$	This relationship describes the ability of the inductor to serve as a conduit between the electric and magnetic domains, making it the true constitutive relationship for an inductor.

## Conclusion

We have developed a kinematic periodic table of fundamental passive elements starting from just the concept of charge in its various states of rest and motion. Neither the magnetism nor electric flux based memristor finds a place in the periodic table due to *any* of the following reasons.

1. The ideal memristor is an unphysical active device.
2. The physical memristor violates Rule 1 by co-occupying a resistor’s slot.
3. The physical memristor violates Rule 2 by needing a time-interval for integration.
4. The physical memristor:
  - is a composite, bounded by the low and high resistance states and
  - requires active hysteresis to switch between the two states.

The chart of fundamental elements from Strukov *et al*., transcribed in Fig. 3(a) becomes untenable when we define and adhere to strict rules for the creation and population of the periodic table of fundamental elements.

Supporters promote that the memristor is fundamental because it cannot be modeled with the traditional *C*, *R* and *L*^19^. We respond that memristors cannot be modeled with *C*, *R* and *L*, not because they are fundamental but because memristors are composite resistors that rely on active hysteresis to switch from low to high resistance or vice versa.

The memristor’s potential for phenomenal computing is in no way diminished by this negative assessment of its qualifications as a fundamental device.

## Methods

We have used the idea that charge is the single fundamental electronic entity that an electrical engineer or scientist works with. Separation of positive and negative charges builds up an electrical potential energy and a non-zero voltage appears. Current, magnetism etc. are a result of various states of motion of charge. This notion was translated into a periodic table of fundamental elements in both the charge-voltage and charge-flux (magnetic) domains. Inspection and discussion shows that the memristor, whether magnetism or electric flux based, cannot exist in this periodic table as a passive fundamental entity.

### Data availability

No datasets were generated or analyzed during the current study.
